# Intranasal Drug Delivery by Nanotechnology: Advances in and Challenges for Alzheimer’s Disease Management

**DOI:** 10.3390/pharmaceutics16010058

**Published:** 2023-12-29

**Authors:** Sayali Dighe, Sunil Jog, Munira Momin, Sujata Sawarkar, Abdelwahab Omri

**Affiliations:** 1 Department of Pharmaceutics, SVKM’s Dr. Bhanuben Nanavati College of Pharmacy, University of Mumbai, Mumbai 400056, India; 2 Indoco Remedies Private Limited, Mumbai 400098, India; 3 The Novel Drug & Vaccine Delivery Systems Facility, Department of Chemistry and Biochemistry, Laurentian University, Sudbury, ON P3E 2C6, Canada

**Keywords:** Alzheimer’s disease, brain targeting, intranasal route, nanocarriers

## Abstract

Alzheimer’s disease, a progressive neurodegenerative condition, is characterized by a gradual decline in cognitive functions. Current treatment approaches primarily involve the administration of medications through oral, parenteral, and transdermal routes, aiming to improve cognitive function and alleviate symptoms. However, these treatments face limitations, such as low bioavailability and inadequate permeation. Alternative invasive methods, while explored, often entail discomfort and require specialized assistance. Therefore, the development of a non-invasive and efficient delivery system is crucial. Intranasal delivery has emerged as a potential solution, although it is constrained by the unique conditions of the nasal cavity. An innovative approach involves the use of nano-carriers based on nanotechnology for intranasal delivery. This strategy has the potential to overcome current limitations by providing enhanced bioavailability, improved permeation, effective traversal of the blood–brain barrier, extended retention within the body, and precise targeting of the brain. The comprehensive review focuses on the advancements in designing various types of nano-carriers, including polymeric nanoparticles, metal nanoparticles, lipid nanoparticles, liposomes, nanoemulsions, Quantum dots, and dendrimers. These nano-carriers are specifically tailored for the intranasal delivery of therapeutic agents aimed at combatting Alzheimer’s disease. In summary, the development and utilization of intranasal delivery systems based on nanotechnology show significant potential in surmounting the constraints of current Alzheimer’s disease treatment strategies. Nevertheless, it is essential to acknowledge regulatory as well as toxicity concerns associated with this route; meticulous consideration is required when engineering a carrier. This comprehensive review underscores the potential to revolutionize Alzheimer’s disease management and highlights the importance of addressing regulatory considerations for safe and effective implementations. Embracing this strategy could lead to substantial advancements in the field of Alzheimer’s disease treatment.

## 1. Introduction

Alzheimer’s disease represents an advanced neurodegenerative condition characterized by compromised cognition, challenges in daily tasks, and difficulties related to learning, speech, and language [1,2]. Projections indicate that by 2050, dementia will impact over 100 million individuals worldwide, with associated costs estimated to escalate to USD 1 trillion in the coming years. Dementia, a prominent manifestation of Alzheimer’s disease, displays age-related progression, doubling approximately every five years past the age of 65 and increasing by about 50% beyond the age of 85. The distinctive molecular features of Alzheimer’s disease encompass the accumulation of Aβ, leading to the formation of senile plaques, excessive tau phosphorylation resulting in neurofibrillary tangles (NFTs), compromised glial function, neuronal inflammation, and irregularities in vascular activity [3,4].

It is also recognized as a protein-conformational disorder (PCD), as the misfolding of neuronal proteins leads to altered conformations that transform soluble forms into insoluble aggregates [5]. AD is acknowledged as a multifactorial ailment, yet current knowledge of the disease highlights NFTs and Aβ plaques as primary contributors to its onset and progression [6]. For decades, research efforts have been directed toward unravelling the biology and mechanisms of the Aβ peptide in AD’s pathogenesis [7]. To simplify the intricate pathology, various hypotheses such as the amyloid cascade hypothesis, tauopathies, and the cholinergic hypothesis [8,9,10,11,12] have been proposed by investigating the disease at both the cellular and molecular levels [13]. Additionally, a mounting body of evidence supports the substantial role of oxidative stress [14,15], neuroinflammation [16], neuron-associated astrocytes, and metal ions such as aluminium in the initiation and advancement of AD [17,18,19,20,21,22]. Brain imaging studies utilizing PET scans in AD patients have revealed heightened levels of activated microglia [23,24,25], along with inflammatory cytokines. Moreover, research has demonstrated that Aβ activates the innate immune response [26,27]. Similarly, dysregulated glutamatergic signaling and the hyperactivation of NMDA receptors result in calcium dysregulation, which is one of the underlying mechanisms that causes AD to progress. Among all of these discoveries, the cholinergic hypothesis and the role of NMDA receptors marked a significant breakthrough in Alzheimer’s disease research, as they form the basis of current conventional pharmacological treatments for AD.

The current available treatments for AD can be categorized into pharmacological interventions targeting altered disease-related neurotransmitters (e.g., acetylcholinesterase inhibitors (AChEIs)such as galantamine and N-Methyl-D-aspartate receptor (NMDA) antagonists such as memantine) and non-pharmacological strategies primarily focusing on behavioural aspects [28]. The elevated level of AChEs in the brains of people with AD prompted researchers to identify AChEIs to substantiate their cholinergic activity, yet research has underscored the significance of both AChE and BuChE in the progression of AD [29]. As a result, there are two categories of cholinesterase inhibitors: non-specific inhibitors that act on both AChE and BuChE; and specific inhibitors that target acetylcholinesterases exclusively or are classified based on the degree and type of inhibition, such as reversible (donepezil, galantamine), irreversible, and pseudo-irreversible inhibitors (rivastigmine).Given the multifaceted nature of the disease, tackling its progression or achieving a cure with a single therapeutic agent is challenging. Consequently, numerous investigations have explored combinations of AChEIs with other agents, such as choline precursors, NMDA antagonists [30], and antioxidants [29]. In this context, several preclinical studies have demonstrated synergistic activity when combining donepezil (AChEI) actions by inhibiting AChEs and memantine (an NMDA antagonist), which execute anti-Alzheimer’s disease action by regulating the Ca^2+^ influx, glutamanergic signalling, etc., leading to overall improved cognition [31,32]. Based on substantial evidence, a fixed-dose combination of donepezil and memantine, known as Namzaric TM, received approval from the FDA in 2014 [33,34]. Despite promising results in providing symptomatic care, these medications have shown inconsistent effects as disease-modifying therapies. Moreover, they can induce serious side effects, such as nausea, diarrhoea, dizziness, and appetite loss [35].

Therefore, the pursuit of novel treatments that alter the course of the disease is currently a top global research priority. The undeniable role of Aβ plaques and tau proteins in the pathology of AD has led research efforts to predominantly focus on these as unique targets for disease-modifying therapies [36,37]. A significant breakthrough in AD research occurred with the recent FDA approval (2021) of the first disease-modifying monoclonal antibody, aducanumab (Aduhelm^®^) [38], which targets Aβ plaques, including both insoluble fibrils and oligomers [39]. In a double-blinded clinical trial, a 1-year infusion of aducanumab demonstrated a controlled reduction in Aβ plaques based on dosage and time [40].These findings were supported by two Phase 3 randomized trials, ENGAGE and EMERGE [41]. However, the accelerated approval of aducanumab was controversial due to safety concerns, and serious side effects such as the development of ARIA, brain oedema, microhaemorrhages, and vertigo, etc., led to its initial disapproval [42,43,44,45]. Despite these concerns, the drug was eventually re-approved as no fatalities were reported. Further, the US FDA mandates post-approval clinical trials to validate the anticipated benefits of aducanumab [46]. Likewise, two humanized monoclonal antibodies, lecanemab (Leqembi^®^) [47] and gantenerumab, obtained FDA approval in 2023 [48].Both of these antibodies demonstrated a high binding affinity to Aβ protofibrils, a potential reduction in Aβ burden, and the deceleration of disease progression in early-stage patients [49]. Further biweekly infusions of lecanemab in Phase 2 trials showcased a time-dependent attenuation of ARIA, with more pronounced occurrences in the ApoE4-positive homozygous population [50,51].In the latter case of gantenerumab, two separate Phase 3 trials (SCarlet RoAD and Marguerite RoAD) were conducted to assess the safety profile and therapeutic efficacy of low-dose subcutaneous gantenerumab [52], and an open-label extension (OLE) study was performed at an escalated dose(up to 1200 mg), which revealed a significant Aβ reduction [53]. Currently, a randomized, double-blind Phase 3 trial, GRADUATE I and II, is underway to evaluate the safety and efficacy of subcutaneous gantenerumab compared to a placebo in early AD populations [54].

Despite promising preclinical results, many Aβ-directed therapies have failed to show efficacy in clinical trials [55]. Consequently, research has shifted towards exploring other potential targets, such as tau proteins and neuroinflammation. This shift has led to the investigation of a wide array of immunotherapies targeting Aβ fibrils and tau proteins for AD treatment, some of which are enumerated in Table 1. However, the efficacy of anti-tau therapy is influenced by factors like the mode of action, existing tau forms, and the epitope and form of tau that spreads to other cells [56]. A growing body of studies indicate discrepancies between pathogeneses, disease severity, and diagnoses, which impacts the success of treatments. Furthermore, the chosen approach for delivering therapeutics to the brain is a pivotal determinant in the success of immunotherapy [57]. Consequently, achieving an efficient and safe delivery of both conventional approved therapeutics and immunotherapies remains a formidable challenge in AD treatment.

**Figure 1:**
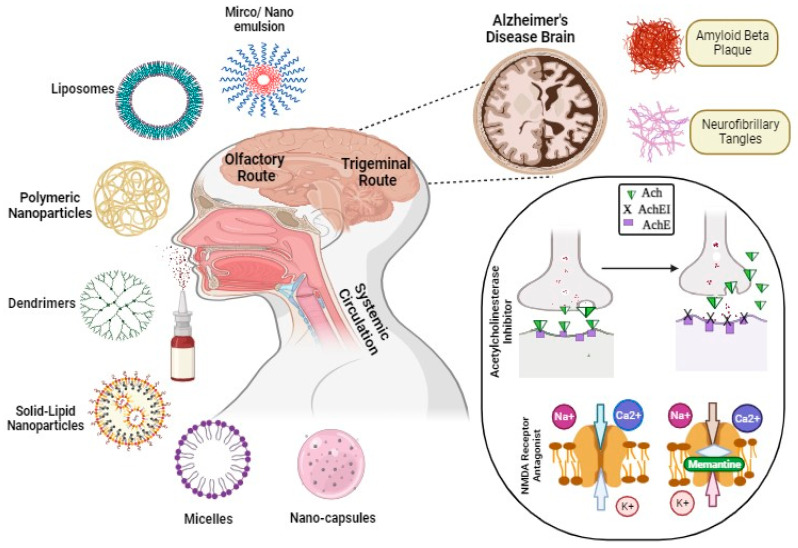
The graphical representation delineates the primary hallmarks of Alzheimer’s disease, elucidates the mechanisms underlying current treatment strategies, and outlines various intranasal treatment approaches for Alzheimer’s disease management based on nanocarriers.

**Figure 2:**
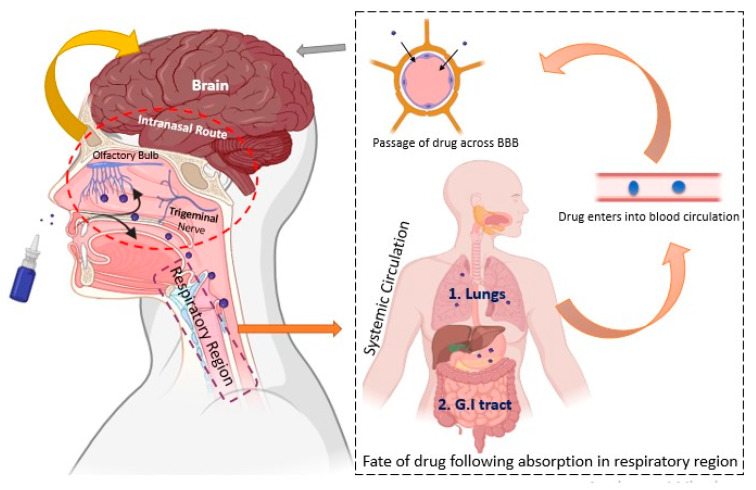
Visual representation illustrating the trajectory of the delivery system after transport through distinct intranasal pathways, namely: olfactory pathway, trigeminal pathway, and respiratory pathway.

Recent advancements in nanotechnology have positioned it as a promising domain for brain targeting, and numerous studies have demonstrated its potential in managing Alzheimer’s disease (AD) [66]. Furthermore, a variety of factors including physiological barriers, brain anatomy, and physicochemical properties significantly impact the therapeutic effectiveness of conventional anti-AD drugs [67]. Thus, adopting a nanocarrier-based delivery approach holds promise for enhancing the efficacy of existing treatments [68]. Loaded with drugs, these nanocarriers elevate the drug concentration in the brain, thus reducing the required dosage and associated side effects [69]. Additionally, nanomedicines contribute to improved stability, biocompatibility, biodegradability, reduced toxicity, an extended half-life, controlled release, and the enhanced solubility of poorly soluble drugs [70].Nanocarriers follow various transport mechanisms to traverse the blood–brain barrier (BBB), including simple diffusion, transcytosis, receptor-mediated endocytosis, and exocytosis [71]. NP diffusion is facilitated through two mechanisms: the first involves stimuli (generated by the “nano-effect” or bioactive substances adsorbed on NP surfaces) mediating the transient opening of tight junctions, followed by diffusion. The second mechanism entails NP adsorption on endothelial cell surfaces, leading to drug release, the creation of a concentration gradient, and the subsequent promotion of diffusion [72]. Moreover, lipid nanoparticles with small molecular weights (<400 Da) and sizes (<100 nm) can effortlessly diffuse through the BBB due to their inherent lipidic nature [73]. Furthermore, active targeting through receptors can be achieved by modifying the surface of nanocarriers with various ligands, such as peptides, polysaccharides, antibodies, and more [74]. This enables tailored nanocarrier systems to achieve specific tissue accumulation in the brain through passive or active targeting mechanisms [75].In spite of such phenomenal characteristics when administered via the conventional route, only 5% of the dose reaches the brain while the remaining 95% accumulates in non-targeted/peripheral tissues, causing potential toxicity to the reticuloendothelial system, etc. Hence, research pipelines have tended toward exploring novel strategies to improve the delivery of nanocarriers to intricate organs, including the brain [76,77,78]. In recent years, intranasal drug delivery has surfaced as a non-invasive, safer, and efficacious alternative to traditional routes of brain targeting [79]. Figure 1 presents various intranasal treatment approaches for Alzheimer’s disease management based on nanocarriers. The potential of the intranasal route of brain targeting is exceptional and can be attributed to unique olfactory and trigeminal pathways that provide direct access to the brain. However, there still exist some anatomical and structural challenges associated with the IN route, e.g., limited volume, mucociliary clearance, etc., which affect the targeting potential [80]. One of the ground-breaking strategies to overcome the aforementioned challenges is integrating nanoscale carriers with the intranasal route of brain targeting. Several studies have demonstrated that nanocarriers administered via the IN route accumulate in a higher concentration at the olfactory bulb and pons, suggesting nanocarriers can readily transverse across the BBB via the intranasal route [80]. Due to the significance of intranasal nanocarriers in brain targeting, diverse polymer-, lipid-, and metal-based carriers have been explored for managing AD [81]. Figure 2 illustrates the trajectory of the delivery system after transport through distinct intranasal pathways. 

## 2. Exploring Nanocarriers for Alzheimer’s Disease Therapy

### 2.1. Polymeric Nanoparticles

A burgeoning and innovative approach to delivering therapeutics to the brain in the context of Alzheimer’s disease (AD) involves the utilization of polymeric nanoparticles. These nanoparticles can be synthesized from monomers or polymers using various polymerization methods [82]. The physicochemical properties of polymeric nanoparticles can be customized according to their intended application. For AD, a diverse range of synthetic polymers (such as PACA and PLGA), natural polymers (including chitosan and alginate), and hybrid polymers have been employed [83]. Diverging from vesicular carriers like liposomes and micelles, polymeric nanoparticles offer distinct advantages such as enhanced stability, reduced drug exposure, and tuneable properties achievable through composition and structural modifications [84]. The traversing of nanoparticles across the blood–brain barrier (BBB) can be facilitated by functionalizing them with ligands, which can occur through various mechanisms: 1. absorbing macromolecules from the bloodstream, enabling interaction with specific receptors (e.g., tween 80) [85]; 2. direct binding to receptors (e.g., lactoferrin) [86]; 3. increasing hydrophobicity and charge (e.g., amphiphilic peptides) [87]; and 4. prolonging circulation time (e.g., PEG) [88]. Additionally, absorptive-mediated transcytosis can be promoted by attaching cationic peptides to the surface of nanoparticles or using cationic polymers (e.g., chitosan) [89]. These cationic nanoparticles engage in electrostatic interactions with negatively charged capillary endothelial cells, facilitating adsorptive-mediated transcytosis transport [90]. However, the exact transport mechanism of nanoparticles remains incompletely understood, and the influence of physicochemical properties on transport remains to be fully elucidated [91].

Upon successful transport, it is subsequently crucial to consider the mechanism of drug release from the carrier. The predominant mechanisms through which polymeric systems achieve controlled release encompass drug diffusion through aqueous pores, matrix diffusion, osmotic-driven release, and erosion mechanisms. Several factors, including the molecular weight, mechanical strength, solubility, nature of the polymer, and glass transition temperature (Tg), affect the drug release profile from polymeric nanoparticles [92]. Various polymers are being investigated for effectively targeting different anti-AD agents, as summarized in Table 2. Despite promising outcomes, clinical applications of polymeric nanoparticles face challenges posed by oxidative stress, cytotoxicity, and genotoxicity, often linked to the quantum dimensions of the nanoparticles [93].

### 2.2. Lipid-Based Nanocarriers

Lipid-based nanocarriers present an innovative avenue for brain targeting, attributed to their lipophilic nature, biocompatibility, biodegradability, and their ability to bypass P-glycoprotein (P-gp) efflux [98]. A significant advantage of lipid nanocarriers lies in their ability to tailor structural properties based on the physicochemical attributes of small drug moieties and excipients. Moreover, the incorporation of lipids as fundamental constituents contributes to achieving distinct controlled release and non-toxic degradation products, in contrast to polymeric nanoparticles, which often exhibit an initial burst release, instability, and toxicity of degradation products [99]. The ease of preparation, avoidance of first-pass metabolism, reduced use of organic solvents, and potential for scale-up further elevate the appeal of lipid nanoparticles over polymeric alternatives [100]. Prominent among lipid nanocarriers for brain targeting are solid lipid nanoparticles (SLNs), nanostructured carriers, and liposomes, largely due to their capacity to circumvent the BBB [101]. Table 3 summarizes various lipid based nanocarriers that have been investigated for targeting drug for effective therapy of Alzheimer’s disease.

Liposomes, a lipid-based vesicular nanocarrier, have versatile applications, including gene delivery, therapeutic administration, and nucleic acid delivery to the brain [109]. Liposomes serve as ideal carriers for gene delivery, benefiting from the incorporation of ionizable or fusogenic lipids, which enhance endosomal escape, target specificity, diminish immunogenicity, and extend circulation time [110]. However, the presence of lipids in liposomes yields a dual-edge characteristic, conferring biocompatibility while also increasing susceptibility to peroxidation and leakage, leading to compromised stability and shelf life. Moreover, challenges such as limited drug loading and entrapment efficiency hinder their clinical application [111]. Therefore, research efforts are directed towards enhancing the stability of existing liposomes and devising novel carriers with an improved stability.

Solid lipid nanoparticles, as the first generation of lipid nanocarriers, were designed to surmount the limitations of liposomes by utilizing lipids to replace the aqueous core, thereby preventing active drug interactions [112]. SLNs also possess the ability to evade the brain’s reticuloendothelial system [113]. The choice of surfactants significantly impacts SLNs’ formation, influencing their particle size, distribution, and targeting efficiency [114]. Some studies have demonstrated increased brain uptake with surfactant-coated SLNs, notably Polysorbate-80 coating, possibly due to the stimulation of endocytosis by transporters such as apolipoprotein E present at the BBB [115]. Additionally, coating SLNs with cationic polymers like chitosan has been shown to enhance drug loading, overcome initial burst release, and improve stability [116]. SLNs have been extensively investigated to enhance bioavailability, BBB transport, and brain targeting for AD management [117].

While SLNs have demonstrated broad applications in brain targeting, nanostructured lipid carriers (NLCs) are preferred from a formulation perspective, offering a high payload due to their imperfect structure, enhanced stability, and reduced risk of drug expulsion [118]. In addition to improved BBB transport, NLCs exhibit a high affinity for Aβ plaques, followed by degradation [119]. NLC surfaces can be tailored through surfactants and ligands like lactoferrin for active targeting [120]. Unlike solid lipid carriers, nanostructured carriers exhibit a dual release mechanism, involving an initial rapid release followed by a sustained release. This characteristic is advantageous for brain targeting [76]. The literature indicates that NLCs can enhance the pharmacokinetic properties and therapeutic efficacy of various anti-AD agents, including donepezil, rivastigmine, antioxidants (e.g., ubiquinone), and ECGCs [121,122,123].

Although lipid-based nanocarriers, particularly LNPs, hold substantial promise for brain targeting, challenges remain in terms of scale-up due to issues such as instability, polymorphism, aggregation, safety concerns, and sterilization-related problems [124].

Nanoemulsions, a biphasic emulsion system, exhibit broad applicability in enhancing bioavailability and targeting across various administration routes [125]. Nanoemulsions offer advantages over microemulsions, such as maintaining globule size regardless of dilution or temperature changes and achieving spherical and smaller globule sizes (<200 nm) [126]. The narrow particle size distribution and inherent lipid nature of nanoemulsions contribute to improved brain uptake across the BBB. Furthermore, they enhance drug stability against degradation, ultimately reducing the required dose and associated side effects [127]. Notably, conventional anti-Alzheimer’s disease drugs like memantine have been delivered to the brain using nanoemulsion formulations, demonstrating enhanced brain uptake with a sustained release of up to 80% [128]. The functionalization of nanoemulsions with ligands, such as shuttle peptides, can further augment uptake and contribute to active targeting [129].

### 2.3. Metal Nanoparticles

Metal nanoparticles have garnered substantial interest due to their distinctive physicochemical attributes and their potential for theragnostic applications in Alzheimer’s disease (AD) management [130]. Various metal nanoparticles, such as gold nanoparticles, silver nanoparticles, iron nanoparticles, and more, have been explored for their anti-Alzheimer’s effects. An intriguing aspect of metal nanoparticles is their inherent ability to permeate the blood–brain barrier (BBB) without requiring additional functionalization, primarily achieved through endocytosis involving both pinocytosis and phagocytosis mechanisms [131,132].

Among these, gold nanoparticles (AuNPs) have captured significant attention owing to their exceptional optical properties, electrical conductance, enhanced stability, and low toxicity. They have demonstrated the potential to counteract memory impairment, as well as inhibit and disaggregate Aβ aggregates [133]. The anti-Aβ properties of AuNPs are influenced by their physicochemical characteristics, such as their size, shape, and charge [134]. Some studies have revealed that rod-shaped, cationic gold nanoparticles exhibit a superior binding affinity to Aβ plaques compared to cube-shaped, anionic gold nanoparticles [135]. Additionally, selenium nanoparticles have demonstrated neuroprotective effects attributed to their reduced toxicity and antioxidant properties [136].

However, while metal nanoparticles exhibit promises as theragnostic tools for AD, studies have also highlighted significant toxicity associated with certain metal nanoparticles, like mercury, aluminium, and copper, and their potential correlation with AD pathogeneses [137]. The primary mechanism underlying this toxicity involves the generation of oxidative stress which damages macromolecules and cells [138]. Consequently, efforts are being directed toward mitigating metal toxicity through various approaches, such as the biogenic synthesis method [139].

Recently, a fusion of metals and organic ligands has led to the formation of “metal-organic frameworks” (MOFs), which offer biocompatibility, stability, improved delivery efficiency, and diagnostic applications [140]. Numerous studies have investigated the role of metal nanoparticles in enhancing brain targeting for AD management, with some of these studies summarized in Table 4.

Advancements in nanocarrier-based delivery systems have ushered in a significant breakthrough in enhancing the clinical effectiveness of treating complex disorders like Alzheimer’s disease. Leveraging their distinctive physicochemical properties and structural attributes, nanocarriers have demonstrated the potential to elevate therapeutic efficacy and enhance the brain uptake of conventional anti-Alzheimer’s drugs. While the solubility and bioavailability benefits offered by nanocarriers are unquestionable, the extent of improvement critically hinges on the chosen administration route [146].

Oral administration is less conducive for brain targeting due to inherent limitations, such as unpredictable or reduced bioavailability, increased dosage requirements and frequency, enzymatic degradation leading to an insufficient drug concentration reaching the brain, and more [147]. Overcoming the BBB and achieving targeted drug delivery have prompted the exploration of various invasive and non-invasive routes [148]. Invasive methods to breach the BBB encompass osmotic, chemical, ultrasound-mediated disruption, intra-cerebro-ventricular, and intrathecal infusions [149]. While effective in conditions like glioblastoma, these approaches entail significant drawbacks, including pathological changes in the brain, perturbed glucose uptake and homeostasis, toxicity to cerebral tissues, and disrupted brain function. Additionally, several of these techniques require high drug doses, potentially leading to toxicity [150,151].

Hence, non-invasive alternatives are under investigation. These include enhancing intracellular transport using transport carriers [152] and inhibiting efflux transporters, although initial inhibitors demonstrated notable toxicity risks [153]. Another strategy involves modifying drug structures to enhance lipid solubility (prodrugs) by limiting polar groups or attaching hydrophilic moieties to lipophilic side chains [154]. While this approach can enhance uptake to some degree, it often necessitates intricate compound engineering.

Further non-invasive methods encompass the Trojan horse approach, chimeric peptides, monoclonal antibody (MAB) fusion proteins, nanoparticle-based delivery, and intranasal delivery [155,156,157]. Each approach presents its own merits and limitations, but combining two or more approaches could potentially yield superior outcomes through dual targeting [158].

Intranasal drug delivery stands out as a well-recognized and established non-invasive strategy for treating various brain disorders [159]. The nasal cavity provides a direct route to the brain through olfactory and trigeminal pathways, while the highly vascularized nasal mucosa enables rapid drug absorption [160]. Enhanced brain targeting via intranasal delivery can reduce necessary dosage levels and minimize exposure to peripheral organs, thus mitigating toxicity [161]. Furthermore, compared to the oral route, intranasal administration offers a rapid onset of action, bypasses first-pass metabolism, and attenuates dose-related side effects [162]. Nonetheless, it is imperative to comprehensively grasp the physiological intricacies of intranasal targeting before formulating a dosage form.

## 3. Transport Mechanisms of Intranasal Route

At present, the treatment of Alzheimer’s disease primarily relies on systemic drug administration, usually in the form of oral or intravenous dosage forms. However, these conventional delivery methods come with several limitations, such as poor bioavailability, extensive first-pass metabolism, a slow onset of action, limited permeability, and restricted access to the brain due to the presence of the blood–brain barrier. In response, the intranasal route of administration has emerged as a promising avenue for addressing various brain-related disorders. The nasal cavity offers a direct pathway for nose-to-brain drug delivery via the olfactory and trigeminal pathways. The highly vascularized nasal mucosa facilitates rapid drug absorption and opens the door for a potential dose reduction through improved brain targeting. While intranasal delivery shows potential as a route for various therapeutic agents, including those for Alzheimer’s disease treatment, a thorough understanding of the physiological aspects of nasal drug delivery is crucial before developing a dosage form.

Brain targeting through the intranasal route predominantly occurs through three pathways: the respiratory pathway (an indirect route), the olfactory pathway, and the trigeminal pathway (a direct route) [163]. Intranasally administered drugs can travel through different pathways, including absorption by the nasal mucosa into the systemic circulation, axonal transport to the olfactory bulb, or direct entry through the trigeminal nerve [164]. Both the olfactory and trigeminal pathways are considered effective and safe routes for delivering active substances to the brain [165]. Gaining a comprehensive understanding of the mechanisms underlying these pathways is essential for devising effective therapeutic strategies for Alzheimer’s disease.

The olfactory neuronal pathway encompasses intra- and extra-neuronal mechanisms [166], spanning the olfactory epithelium, olfactory bulb, and lamina propria. Administered drugs reach the olfactory bulb from the olfactory region through a transcellular mechanism [167]. Additionally, various mechanisms such as paracellular transport, transcytosis, and diffusion, as well as the involvement of efflux transporters [168], can come into play based on the physicochemical properties of the drug. The olfactory bulb serves as a direct conduit for distributing the drug to different brain regions, including the piriform cortex, hypothalamus, and amygdala [150].

Another significant route for delivering active agents to the brain is the trigeminal pathway [169]. These nerves are present in the nasal epithelium of the respiratory region and extend to the brain via the pons, connecting with the olfactory bulb [170]. Within intranasal delivery, the ophthalmic and maxillary divisions of the trigeminal nerves play a pivotal role, as neurons in these areas directly traverse the nasal mucosa [150]. The segment of the trigeminal nerve that passes through the cribriform plates may contribute to drug delivery to the forebrain [171]. While this pathway is as equally significant as the olfactory pathway for delivering drugs to the anterior and other important brain regions, distinguishing the exact contribution of each pathway can be challenging [150].

Mucus within the nasal cavity plays a vital role in drug delivery and absorption. Mucin, a protein present in mucus, has the potential to bind with solutes, thereby influencing the diffusion process. Multiple mechanisms, including paracellular and transcellular routes [172], are involved in nasal delivery and absorption through the mucosa.

Intranasal drug delivery for neurological diseases has garnered significant attention. However, achieving targeted drug delivery to specific areas of interest remains a challenge due to a multitude of factors, encompassing the drug’s physicochemical properties, experimental conditions, and anatomical and structural characteristics [173]. Thorough investigations into and control of the therapeutic’s physicochemical attributes, including its nature, molecular weight, lipophilicity, shape, and size, are essential for successful formulation development via the intranasal route [174]. For instance, Huang et al. discovered that the ester form of L-tyrosine exhibited greater nasal absorption than that of L-tyrosine [175]. It has also been observed that nasal absorption is enhanced with lower-molecular-weight, cyclic molecule shapes [175]. Nevertheless, when the molecular weight of the active component surpasses 300 Da, permeability challenges may arise [176]. In the context of brain targeting, effective drug deposition within the olfactory epithelium hinges on dosing conditions, including head positioning, the administration technique, and the volume delivered [177]. Alongside dosing considerations, physiological factors such as the blood flow, enzyme activity, and mucociliary clearance of the nasal cavity can impact the absorption, therapeutic stability, and residence time.

Kushwaha et al. (2011) established a direct relationship between absorption and residence time, inversely linked to mucociliary clearance [175]. To surmount challenges associated with physiological and physicochemical factors, diverse strategies have been explored. These include the utilization of varied formulations (like dendrimers and vesicular systems) and permeation enhancers that modify the nasal cavity’s epithelial barrier. The nasal delivery of peptides, such as insulin, was limited due to degradation and a short half-life. To address these concerns, researchers delved into the prodrug approach [178], which not only provides protection but also enhances lipophilicity [179]. The incorporation of absorption enhancers has also proven effective in augmenting nasal delivery and targeting [180]. For instance, Chavanpatil et al. [181] examined the use of hydroxypropyl β-cyclodextrin, sodium deoxycholate, sodium caprate, sodium tauroglycocholate, and EDTA as penetration enhancers for the intranasal delivery of acyclovir. However, these approaches are not without drawbacks, including potential nasal toxicity, nasal mucosa damage [182,183], and limited success in breaching the BBB and precisely localizing therapeutics in the brain.

Therefore, a pressing need exists for a delivery system that can effectively traverse central nervous system barriers and guide the active ingredient to its intended target site without disrupting the physiology and structure of the nasal epithelium or the blood–brain barrier. Nanocarrier-based drug delivery systems present a promising alternative to traditional intranasal delivery methods [166,184,185]. Polymers, metal- and lipid-based particulate systems, vesicular carriers, and miscellaneous carriers such as nanoemulsions, nanosponges, dendrimers, and quantum dots are extensively explored nanocarrier-based platforms for intranasal drug delivery in the context of Alzheimer’s disease. The following section delves into various nanocarrier approaches reported for brain targeting via the intranasal route, aiming to effectively manage Alzheimer’s disease.

## 4. Intra-Nasal Nanoparticulate System for Alzheimer’s Disease Treatment

### 4.1. Nanoparticle-Based System

The utilization of nanoparticle-driven drug delivery has demonstrated its effectiveness in enhancing the absorption of nasal therapeutics. By encapsulating the drug within nanoparticles and safeguarding it from enzymatic degradation, therapeutic concentrations are elevated at the target site [186,187]. While the blood–brain barrier typically restrains particles exceeding 200 nm in size, nanoparticles with dimensions smaller than 200 nm can traverse the olfactory pathway [188]. Polymeric and metal nanoparticles have garnered attention for their potential in managing Alzheimer’s disease, offering several advantages such as a heightened loading capacity, degradation protection, enhanced stability, precise targeting, a reduction in dosage, and the potential for affinity enhancement for Aβ proteins, a hallmark of AD [189]. Furthermore, the surface modification of these nanoparticles can enhance their interaction with Aβ proteins.

Biodegradable and biocompatible polymers like chitosan, poly D, L-lactic-co-glycolic acid (PLGA), and polyvinyl alcohol (PVA) have been harnessed for intranasal drug delivery due to their controlled-release properties [190]. Chitosan, with its bio-adhesive nature, low toxicity, resistance to mucociliary clearance, and ability to prolong nasal residence time in the olfactory region, stands out as a preferred choice for nanoparticle formulations. This prolonged residence is attributed to interactions between the chitosan polymer’s polysaccharide moiety and the corresponding saccharide groups of the nasal mucosa. Chitosan also has the capacity to perturb intercellular tight junctions, thereby enhancing drug permeability.

Studies have delved into chitosan-based nanoparticles for intranasal drug delivery targeting Alzheimer’s disease. For instance, Wilson et al. developed chitosan nanoparticle-encapsulated sitagliptin and found a five-fold increase in the sitagliptin concentration compared to that of free sitagliptin. Furthermore, sitagliptin-loaded nanoparticles exhibited enhanced brain accumulation, potentially due to chitosan’s ability to modulate tight junctions [191]. In another study, Kandil et al. administered intranasal galantamine–chitosan complex nanoparticles to Wistar rats. This intervention led to reduced levels of MDA (malondialdehyde) and tumour necrosis factor-α in the brain extracts of nanoparticle-treated subjects in comparison to those of the control group. Conversely, higher levels of superoxide dismutase and glutathione were observed in the group treated with the galantamine–chitosan nanoparticles [192].

Zhang Li et al. conducted an in vitro/in vivo correlation (IVIVC) comparison between intranasally administered curcumin-loaded chitosan-coated PLGA nanoparticles and curcumin–hydroxypropyl-beta-cyclodextrin (HP-β-CD) inclusion complexes. The curcumin–HP-β-CD complex exhibited an improved cellular uptake and reduced cytotoxicity and demonstrated an antioxidant effect at a 20µM concentration in BV-2 cells, as compared to that of the curcumin–chitosan-PLGA nanoparticles [193].

Pawar et al. observed an enhanced uptake and reduced nasal clearance in glycol- and chitosan-coated PLGA nanoparticles. The glycol-coated nanoparticles displayed a superior uptake and nasal retention time compared to those of the chitosan-coated PLGA nanoparticles, potentially attributed to their surface charge density and polymer molecular weight [194].

Lastly, Sunena et al. evaluated the in vivo pharmacodynamics of intranasally administered galantamine-loaded thiolated chitosan nanoparticles. Their results underscored the significant delivery advantage of intranasal galantamine–chitosan nanoparticles for oral and nasal routes, highlighting the therapeutic superiority of intranasal administration [195].

The therapeutic potential of piperine (PIP), an alkaloid with cognitive improvement properties, is hindered by its poor aqueous solubility and low bioavailability, necessitating a high-dose regimen. In response, Elnaggar et al. devised a solution by developing intranasal chitosan nanoparticles (CS-NPs) encapsulating PIP, utilizing the ionic gelation technique. These CS-NPs exhibited a spherical morphology with optimal attributes, including a particle size of 248.50 nm, PDI of 0.24, zeta potential of +56.30 mV, and encapsulation efficiency (EE) of 81.70%. Their controlled-release behaviour was evident, with a 92% release achieved by the 24h mark. Comprehensive evaluations confirmed the safety of CS-NPs regarding nasal irritation and brain toxicity. Notably, PIP-NPs matched the effectiveness of standard donepezil injections in enhancing cognitive function, while displaying a remarkable 20-fold reduction in effective dosage compared to the conventional oral dosage. These nanoparticles also exhibited a dual mechanism involving anti-apoptosis and anti-inflammatory effects [196].

Similarly, Fazil et al. employed a similar approach to prepare nasal chitosan nanoparticles loaded with rivastigmine (CS-RHT NPs). Their characterization encompassed parameters like the zeta potential (ZP), particle size, PDI, and %EE. The brain-targeting capability of placebo NPs was assessed using rhodamine-123-based laser scanning microscopy. Pharmacokinetic and distribution investigations revealed a higher brain concentration of rivastigmine with CS-NPs (i.n.) (966 ± 20.66 ng mL^−1^; tmax of 60 min) compared to that of an intranasal drug solution (508.66 ± 22.50 ng mL^−1^; tmax of 60 min) or the intravenous administration of CS-NPs (387 ± 29.51 ng mL^−1^; tmax of 30 min). The drug transport efficiency of the CS-RHT NPs via nasal administration reached 355 ± 13.52%, with the direct transport percentage being approximately 71.80 ± 6.71%. An examination of the brain/blood ratio indicated the highest ratio for the CS-RHT NPs via the intra-nasal administration. Additionally, the study demonstrated the higher permeability of CS-RHT NPs compared to that of the pure drug solution. Overall, these findings underscored the brain-targeting potential of chitosan nanoparticles administered via the intranasal route [197].

A wide range of synthetic polymers, including poly(L-lactide-co-glycolic) acid, poly (lactic acid), and poly (glycolic acid), have been extensively explored for delivering drugs to the brain through the nasal route. The modification of these polymeric nanoparticles using compounds such as PEG (polyethylene glycol) or poloxamers can enhance drug loading, stability, and penetration across the nasal mucosa [195]. Musumeci T. et al. advanced this concept by developing PLGA nanoparticles and NLC-based nanosystems for adsorbing a neutralizing monoclonal antibody targeting TNF-related apoptosis-inducing ligand (TRAIL). Pharmacokinetics and dynamics studies in an AD mouse model demonstrated a high entrapment efficiency (99%) for both formulations, as confirmed by an ELISA. Notably, the intranasal administration of the antibody–nanocarrier complex led to significantly higher brain levels compared to those of the free anti-TRAIL antibody [198].

In a separate investigation, Yu Su et al. devised PEG-PLA nanoparticles loaded with miR132, a crucial molecule for sustaining neuronal survival in the brain. However, due to miRNA’s net anionic charge and low solubility in aqueous media, bare miRNA molecules are prone to rapid degradation or mucosal elimination following nasal administration. Thus, the quest for a carrier that ensures safety, an enhanced stability, and the target specificity level remains. The amalgamation of polylactic acid (PLA) and polyethylene glycol (PEG) generates a core-shell structure in aqueous environments, bolstering nasal permeability while diminishing mucociliary clearance. Animal studies have yielded augmented expressions of SYN and PSD-95, along with the inhibition of neuronal cell apoptosis in peripheral nerve cells and the cerebral cortex, signifying the neuroprotective effect of PLGA nanoparticles [199].

A comparison between intranasal curcumin- and bismethoxycurcumin-loaded PLGA nanoparticles showcased curcumin’s superior anti-inflammatory potential, interacting with molecular targets like amyloid peptide plaques and the cyclooxygenase2 enzyme, responsible for inflammatory reactions within the disease. Nanaki et al. constructed hybrid nanoparticles for nose-to-brain galantamine delivery, for which PLGA nanoparticles exhibited a greater uptake through olfactory unsheathing cells than that of PLA nanoparticles. Successful brain targeting was indicated by strong fluorescence in the hippocampus post intranasal administration, with an observed acceptable level of safety and no toxicity [200].

Protamine-coated PLGA nanoparticles within a Carbopol gel were formulated by Shamarekh et al. for Tacrine brain targeting via intranasal administration. This nanocomposite gel displayed higher Cmax and AUC values after 0–12h in the brain compared to those of i.v. and i.n. drug solutions. A histopathological analysis indicated no damage, suggesting their potential for neurodegenerative disease treatment [201]. Meng et al. developed lactoferrin-functionalized intranasal PLGA nanoparticles modified with N-trimethylated chitosan for effective Huperzine A brain targeting. In vivo imaging showcased prolonged brain fluorescence, with successful targeting evident in the olfactory bulb, cerebellum, cerebrum, and hippocampus following the nasal nanoparticles’ administration [202]. To enhance targetability and minimize mucociliary clearance, researchers have explored nanoparticle surface modifications with specific ligands, which demonstrate superior targeting compared to that of unmodified nanoparticles.

The field of nanomedicine in Alzheimer’s disease (AD) therapy has been a burgeoning area of exploration, particularly in the realm of metallic nanoparticles (NPs) for BBB-targeted delivery. However, the use of metallic NPs is hampered by chemical synthesis methods. Nonetheless, cerium, gold, selenium, and iron metallic NPs have demonstrated potent anti-AD capabilities, finding applications in theranostics, gene delivery, and stimulus-responsive therapies like photothermal treatments for diverse diseases, including cancer. Gold nanoparticles, particularly relevant for crossing the BBB, are being investigated for theranostic AD management. Bastus et al. [203] engineered gold nanoparticles targeting and solubilizing amyloid fibrillar aggregates, indicating their potential for dissolution via microwave-generated thermal energy. Controlled binding with the target through the energy input was established. While promising, exclusive AuNP targeting is imperative to mitigate cytotoxicity associated with amyloid beta oligomer species. Kogan et al.’s non-invasive investigation and amyloid beta aggregate manipulation technique seem advantageous for AD therapy [204]. Moreover, metallic nanoparticles have been explored for diagnostic purposes in detecting β-amyloid plaques in animal models.

Resveratrol, a promising neuroprotective stilbenoid, has the potential to enhance cognitive function in Alzheimer’s disease. However, its clinical efficacy is hindered by its extensive metabolism and poor bioavailability. To overcome these limitations, Salem et al. designed resveratrol-loaded transferosomes and nanoemulsions, incorporating gold nanoparticles (GNPs) for an improved delivery. Various physicochemical properties were assessed, along with dynamic studies such as water maze tests, to analyse spatial memory recovery. The results revealed memory improvements in all treated groups, with the transferosome–GNP gel group matching the normal group. Notably, the transferosome–GNPs exhibited enhanced permeation (81.29±2.64%) and symptom alleviation, with increased gold nanoparticle accumulation [205].

Iron oxide nanoparticles, another category of metal nanoparticles, are widely employed in AD therapeutic management. Zhang et al. devised super paramagnetic iron oxide NPs (SPIONs) modified with1,1-dicyano-2-[6-(dimethylamino)-naphthalene-2-yl] propene carboxyl. This disease model displayed a reduced signal strength in the hippocampal region [206]. Mahmoudi et al. explored the influence of SPIONs’ surface charge and coating thickness on beta amyloid fibrillary dynamics, revealing a direct correlation between the SPION concentration and fibrillation rate. Positively charged SPIONs induced fibrillation at lower concentrations compared to neutrally/negatively charged ones. Leveraging the magnetic properties of amyloid beta fibrils, FDA-approved AD drugs can be coupled with SPIONs or similar metal nanoparticles for targeted intranasal delivery [207].

Addressing the reactive oxygen species (ROS) concentration in the brain represents another crucial AD treatment avenue. Selenium (II), sodium selenite (IV and VI), are potent ROS inhibitors, pivotal in curbing oxidative stress and cellular cytotoxicity. Selenium- and selenite-containing nanoparticles have biomedical applications [208]. Yin et al. synthesized sialic acid (SA)-functionalized selenium (Se) nanoparticles, further linked with substitute peptide-B6 peptide (B6-SA-SeNPs). These nanoparticles showcased enhanced BBB transport, promising a nanomedicine-based strategy for AD modification. Uptake studies and transport capability assessments using cerebral endothelial cells (bEnd.3) and inductively coupled plasma atomic emission (ICP-AES) highlighted B6-SA-SeNPs’three-fold higher uptake compared to that of SA-SeNPs. The transwell method and PC12 co-culture models demonstrated the B6-SA-SeNPs’ superior transport ability. These findings indicate B6 peptide’s potential in enhancing brain delivery, suggesting B6-SA-SeNPs as a favourable platform, particularly for intranasal AD treatment.

Metal nanoparticles present a versatile platform for the intranasal targeting of various therapeutic agents in AD management. Ongoing research endeavours focus on harnessing green-chemistry-based synthesis methods to optimize these nanoparticles for future AD treatments.

### 4.2. Lipid Nanocarriers

Lipid nanocarriers, consisting of solid lipid matrices (SLNs) or combinations of solid lipid and oil matrices (NLCs), have garnered significant attention as versatile delivery systems. These nanocarriers offer benefits such as prolonged retention, reduced clearance, enhanced solubilization and permeation, improved stability, and compatibility within the nasomucosal region. Researchers have extensively explored SLNs and NLCs for intranasal delivery, showcasing improved brain-targeting efficacy. Solid lipid nanoparticles (SLNs) and nanostructured lipid carriers (NLCs) represent lipid-based nanocarriers that excel in delivering both hydrophobic and hydrophilic drugs [209].

Addressing the limitations of risperidone, an anti-psychotic drug commonly used to treat Alzheimer’s-related agitation, Patel et al. engineered solid lipid nanoparticles (RSLNs) using Compritol 888 ATO and Pluronic F-127. These RSLNs exhibited a high entrapment efficiency (59.65% ± 1.18%) and a narrow PdI of 0.148 ± 0.028, indicating formulation stability. Pharmacodynamic assessments using hindlimb retraction time (HRT) in a mouse model demonstrated the superior antipsychotic potential and brain targeting of RSLNs compared to risperidone solution (RS) and a control. The intranasal administration of RSLNs yielded a brain/blood ratio 10-fold higher than that of their intravenous administration, highlighting improved brain concentration [210]. Deepshi et al. utilized a solvent evaporation diffusion method to design rivastigmine tartrate-loaded SLNs, achieving optimized particle size, entrapment efficiency, and drug content. These rivastigmine-loaded SLNs showcased sustained release and improved ex-vivo nasal mucosa flow and diffusion coefficients compared to rivastigmine solution [211]. Similarly, Yasir et al. created donepezil-entrapped solid lipid nanocarriers using glyceryl behenate, exhibiting enhanced targeting potential and improved brain bioavailability [212].

The surface modification of SLNs, akin to polymeric nanoparticles, enhances their target specificity [78]. Yusuf et al. explored surface-modified SLNs for the enhanced bioavailability and brain targeting of piperine. Surface-coated SLNs demonstrated reduced superoxide dismutase values and cholinergic degradation, with a sustained brain concentration and improved bioavailability compared to those of free drug [213]. Saini et al. incorporated ferulic acid into SLNs, enhancing their permeability across lipophilic barriers, and further surface-modified the SLNs with chitosan. The chitosan-coated SLNs showcased a superior drug concentration in the brain, improved cognition, and improved biochemical factor levels in the cortex and hippocampus [214].

While solid lipid nanoparticles (SLNs) have shown potential, their limitations have led to the emergence of nanostructured lipid nanocarriers (NLCs). Anand et al. developed NLCs loaded with rivastigmine hydrogen tartrate for dementia treatment. The NLCs displayed controlled release, enhanced penetration, and decreased acetylcholinesterase expressions, suggesting their potential for Alzheimer’s management [215].

In the realm of Alzheimer’s therapy, lipid nanocarriers hold great promise, offering a transformative approach to drug delivery and targeting within the brain.

The pioneering work of Musumeci et al. [198] aimed to surmount challenges in Alzheimer’s disease (AD) treatment, including high dosage regimens and low transport efficiency. To achieve this, they devised nanostructured lipid carriers (NLCs) through a phase inversion technique without organic solvents (the PIT method). The NLCs were then coated with TRAIL and subjected to freeze-drying using glucose as a cryoprotectant. Immunofluorescence studies on 3xTg-AD and wild-type mice demonstrated that NANO-A and NANO-B complexes, upon being injected intranasally, effectively traversed the BBB of 3xTg-AD mice. This showed successful TRAIL targeting, known to be abundant in hippocampal inflammatory sites. Blocking TRAIL yielded cognitive enhancements and the halting of disease progression and brain degeneration.

In a quest to enhance brain targeting and nasal retention, Vavia et al. [216] delved into an in-situ gel loaded with rivastigmine nanostructured lipid carriers (NLCs). The incorporation of stearylamine (SA) into the NLCs facilitated nasal retention by overcoming mucociliary drainage. Pharmacokinetic and distribution studies revealed NLCs’ sustained release, improved brain penetration, and BBB penetration. This led to cognitive recovery in amnesic mice through intravenous and intranasal administration [216]. Similarly, Jojo et al. devised intranasal pioglitazone NLCs using the micro-emulsion method. The optimized NLCs exhibited increased permeability, flux, and permeability coefficients compared to those of a drug solution. In vivo studies showcased elevated brain/blood ratios, demonstrating the potential of NLCs in clinical AD management via intranasal administration [217].

Moreover, lipid nanoparticles, including solid lipid nanoparticles (SLNs) and NLCs, have demonstrated efficacy as effective carriers for brain-targeted drug delivery. The ingenious utilization of lipid nanocarriers holds immense promise in revolutionizing Alzheimer’s treatment. Through ingenious engineering and innovative delivery strategies, these nanocarriers pave the way for targeted and enhanced drug delivery to the brain, offering renewed hope in the battle against this debilitating disease.

Recent studies have demonstrated the potential of liposomal formulations in revolutionizing Alzheimer’s disease (AD) treatment. In the study by Li et al. (2022) [163], encapsulating Hydroxy-α-sanshool (HAS) within liposomes led to a superior targeting efficacy compared to that of free HAS. Liposomes, owing to their versatility, are capable of encapsulating hydrophilic, hydrophobic, and amphipathic therapeutics. However, overcoming challenges posed by limited blood–brain barrier (BBB) penetration and oral bioavailability is essential for effective AD drug delivery. To tackle this, Rompicherla et al. [218] compared intranasal rivastigmine-loaded liposomes to PLGA nanoparticles. Their results highlighted that liposomal formulations exhibited rapid action and higher concentrations, achieving notable acetylcholinesterase inhibition in plasma and brain homogenate samples. Sokolik VV et al. conducted a comparative analysis between solubilized and liposomal curcumin formulations in an AD model [219].

Curcumin, renowned for its anti-inflammatory properties and potential in reducing Alzheimer’s symptoms, has faced limitations due to its stability and low bioavailability. Overcoming these challenges, intranasal liposomal curcumin displayed enhanced cognitive responses and a greater reduction in cytokine biomarkers, offering a promising avenue for AD treatment [79]. Galantamine hydrobromide, an AD-approved drug, has shown adverse effects when administered through oral and parenteral routes. Seeking an alternative, Li et al. explored an intranasal galantamine hydrobromide (GH)-loaded flexible liposomal formulation. Characterized by highly elastic fluid membranes, flexible liposomes are optimal for efficiently delivering hydrophilic compounds across cell membranes. GH-loaded flexible liposomes demonstrated favourable characteristics, including size and zeta potential. Pharmacokinetic studies indicated superior brain concentrations for formulations administered nasally, with flexible liposomes showing the highest concentration [220].

Furthermore, liposomes have shown great potential as carriers for neurotrophic factors, attributed to their cellular uptake enhancement, lipophilicity, and degradation protection. Cationic liposomes, particularly, have displayed improved protein passage across the nasal epithelium. Migliore et al. developed cationic liposomes loaded with ovalbumin (OVAL), which exhibited persistent brain delivery, highlighting their viability for protein transport [221].

The therapeutic potential of liposomes extends to targeting H102 peptide, which cleaves β-sheets. Zheng et al. developed H102-peptide-based liposomes that demonstrated enhanced brain penetration and reduced degradation, significantly improving spatial memory and enzyme activities in AD-induced rat models [222]. Similarly, Yang et al. explored rivastigmine-loaded liposomes modified with PEGylated poly-arginine CPP derivatives to enhance stability and brain targeting through improved transcytosis [223]. Another strategy by El-Helaly et al. involved introducing a positive charge using dodecyl dimethyl ammonium bromide to maintain stability. Further coupling with PEGylated lipids yielded stable electrostatic stealth long-circulating liposomes, with an increased drug concentration observed in both plasma and the brain [224]. Collectively, these recent studies underscore the potential of liposomes in enhancing Alzheimer’s treatment. Their versatility, stability improvement, and targeted delivery capabilities make them a promising tool in the fight against this debilitating disease.

Arumugam and colleagues ventured into the realm of Alzheimer’s disease (AD) treatment by developing liposomes incorporating rivastigmine. They embarked on a comparative study to discern rivastigmine concentrations in plasma after administering free drugs via oral and nasal routes, orally administered liposomes, and liposomes delivered intranasally. Intriguingly, intranasal liposome administration displayed a remarkable five-fold increase in the area under the curve (AUC) compared to that of orally administered free drugs, and a three-fold rise compared to that of intranasal free drug administration. Furthermore, rivastigmine-loaded liposomes (IN) exhibited a notable 5.6-fold surge in brain concentration and a prolonged half-life (T_1/2_) compared to those of free drug solutions via the nasal and oral routes. This enhancement in absorption can be attributed to effective brain targeting facilitated through the nasal olfactory pathway, with the physicochemical attributes of the drug also playing a pivotal role in breaching the BBB [225].

In addition to the targeting strategies discussed earlier, liposomal carriers can be harnessed with Aβ targeting ligands or brain-penetrating peptides for heightened brain-specific delivery. A new avenue lies in multifunctional liposomes, catering to both therapeutic and diagnostic roles. Mourtas et al. delved into this frontier, crafting DPS–curcumin surface immobilized nanoliposomes for AD treatment. These nanoliposomes exhibited a dual functionality: labelling Aβ deposition with a high efficiency and instigating the inhibition of amyloid beta-42 aggregates. Intriguingly, these multifunctional nanoliposomes could switch between activated and inactivated states, granting them a theranostic capability [226].

Indeed, multifunctional nanoliposomes are gaining attention from various researchers for their potential in brain targeting and the management of Alzheimer’s disease. Table 5 and Table 6 provide an overview of research endeavours concerning polymeric nanoparticles, lipid nanoparticles, and liposomes in the context of Alzheimer’s disease treatment.

### 4.3. Nanoemulsions and Microemulsions

Nanoemulsions are a specialized drug delivery system composed of two non-miscible phases held together by surfactants, resulting in a stable and uniform solution. These formulations typically range in size from 20 to 200 nanometres [123]. Intranasal nanoemulsions have shown promising results in experimental studies, allowing for the direct delivery of small molecules to the brain. This approach addresses challenges related to poor aqueous solubility, limited bioavailability, degradation, and a slow onset of action. The addition of mucoadhesive polymers can prevent the rapid nasal clearance of nanoemulsions [241]. However, these systems are kinetically stable and require a high amount of energy for manufacturing. In contrast, microemulsions (MEs) are pseudo-ternary formulations comprising oil, aqueous media, surfactants, and co-surfactants, forming spontaneously and remaining thermodynamically stable. AnME system usually has a size range from 10 to 100 nm, enabling passive targeting. Both nanoemulsions and microemulsions are biodegradable, biocompatible, and display nanometric sizes. Nevertheless, these formulations can experience sedimentation, creaming, and Ostwald ripening. Proper formulation design can lead to the creation of stable nanoemulsions and microemulsions for extended periods [242]. This section discusses several experimental studies involving the intranasal delivery of Alzheimer’s therapeutics using nanoemulsions and microemulsions.

Atinderpal et al. developed a nasal nanoemulsion containing memantine through a combination of pressure homogenization and ultrasonication. The resulting nanoemulsion’s average size, zeta potential (ZP), polydispersity index (PdI), and entrapment efficiency (% EE) were characterized. In vitro diffusion studies conducted in simulated nasal fluid (SNF) at a pH of 5, phosphate buffer saline (PBS) at a pH of 7.4, and artificial cerebrospinal fluid (ACSF) at a pH of 7.3 demonstrated 80%, 60%, and 40% drug release after 6 h, respectively. The prepared nanoemulsion exhibited first-order release kinetics in SNF and adhered to the Peppas kinetic model in PBS and ACSF. The nanoemulsion exhibited a strong antioxidant potential in FRAP and DPPH assays and displayed a higher reducing potential, which is beneficial for Alzheimer’s treatment. In vivo studies using radiolabelled memantine revealed the highest radioactivity percentage in the brain after intranasal administration. Biodistribution studies and gamma images indicated direct nose-to-brain targeting across the blood–brain barrier (BBB) [128].

The study by Kaur et al. demonstrated the brain-targeting potential and antioxidant activity of intranasal nanoemulsions. Specifically, a technetium pertechnetate (99mTc) labelled donepezil nanoemulsion exhibited successful intranasal brain delivery, as confirmed by scintigraphy imaging. This nanoemulsion showed no adverse effects on cell morphology but displayed dose-dependent cytotoxicity and radical scavenging activity percentage (%RSA) [243].

Furthermore, nanoemulsion systems have exhibited versatility in enhancing brain-targeting efficacy for a wide range of molecules, including poorly soluble drugs such as osthole and resveratrol. For instance, Song et al. formulated a nasal nanoemulsion of osthole, a natural coumarin with potential therapeutic properties. The resulting OST-NE formulation demonstrated significant improvements in spatial memory, decreased cholinesterase activity, increased anticholine content, and neuroprotective effects in mouse models, rendering it a promising option for Alzheimer’s therapy [244].

Similarly, Kota et al. developed a coconut oil-based resveratrol nanoemulsion, and Vasdev et al. formulated a low-energy nanoemulsion using rosemary oil and donepezil for Alzheimer’s treatment. The safety of these nanoemulsions was confirmed through ex-vivo mucosal ciliotoxicity and permeation studies. The low energy requirement of these formulations suggests their potential scalability [245].

Comparative pharmacokinetic studies between a nanoemulsion and suspension, as conducted by Kotta et al., revealed that the nanoemulsion exhibited a higher maximum concentration (Cmax), a shorter time to reach maximum concentration (Tmax), and a larger area under the curve (AUC) compared to those of the suspension, in terms of both plasma and brain distribution. These findings indicate the potential of the developed nanoemulsion as a suitable candidate for targeted drug delivery to the brain [246].

In addition to passive brain targeting, ligand-modified nanoemulsions have been explored for active targeting to the brain. For instance, Jiang et al. optimized a lactoferrin-loaded HupA intranasal nanoemulsion, demonstrating enhanced brain uptake through specific carriers and transcytosis. An in vivo analysis confirmed its successful delivery to the central nervous system, signifying its potential for Alzheimer’s treatment [247]. Recent research efforts have shifted towards exploring the brain-targeting ability of thermodynamically stable dispersion systems like microemulsions. Wen et al. developed an ibuprofen-based microemulsion for managing Alzheimer’s, resulting in a significantly increased brain uptake compared to that of intravenous and oral administrations of ibuprofen. Additionally, Zussy et al. demonstrated that an intranasal microemulsion of nanovectorized docosahexaenoic acid (DHA) improved cognitive ability and reduced tau phosphorylation in AD mouse models [248,249]. Various targeting approaches using microemulsions have been investigated to enhance brain uptake and therapeutic efficacy. For instance, Chen et al. formulated a dual-responsive intranasal microemulgel for the delivery of Huperzine A, exhibiting significantly improved drug exposure in the brain. Another study by Khunt et al. utilized omega-3 fatty acids and butter oil for the targeted delivery of donepezil hydrochloride in a microemulsion formulation via the intranasal route, achieving a superior bioavailability compared to that of the solution [250,251].

Furthermore, Shah et al. conducted a comparative study between a plain microemulsion (ME) and a chitosan-based bioadhesive microemulsion (MME) for the intranasal delivery of rivastigmine. Their results showed that the MME exhibited higher diffusion through the nasal mucosa and an increased concentration of the therapeutic agent in the brain, surpassing the performance of the ME and the solution [252].

In a separate study, Pathak et al. developed a mucoadhesive microemulsion of nimodipine using Carbopol 934. This formulation demonstrated a rapid burst release followed by a sustained release, leading to an increased concentration of the therapeutic agent in the brain [253]. Collectively, these studies underscore the potential of intranasal nanoemulsions and microemulsions as feasible, cost-effective, and scalable approaches for delivering both synthetic and natural treatments for Alzheimer’s disease. The summarized research findings are presented in Table 7.

### 4.4. Miscellaneous Nanocarriers

Nano suspensions represent a promising approach for intranasal drug delivery, especially for poorly soluble agents, by utilizing surfactant-stabilized small-scale dispersions. These systems retain their crystalline structure, have an enhanced drug loading capacity, and can be engineered to avoid phagocytosis for targeted delivery [259].

The challenges of curcumin, a potent neuroprotective compound against Aβ plaques, which include its rapid metabolism and poor absorption, have been addressed by Dibaei et al. They developed a surface-engineered nano-suspension of curcumin using stabilizers such as D-α-tocopheryl polyethylene glycol 1000 succinate and Tween 80. Through high-pressure homogenization and probe sonication, nanocrystals were formed. Their study reported enhanced brain concentrations with the Tween 80-coated curcumin, indicating better ApoE absorption than that of the TPGS-coated NS. However, the TPGS-NS exhibited a higher brain distribution than that of the plain curcumin solution [260]. Bhavna et al. employed an ionic cross-linking technique to create a chitosan-based intranasal nanosuspension of donepezil. The nanosuspension exhibited a size range of 150–200 nm with a PDI of 0.341. Safety evaluations showed no toxicity and no mortality in vivo. Additionally, a higher fraction of donepezil was detected in the brain (147.54 ± 25.08 ng/mL) at a 0.5 mg/mL dose. These findings underscore nanosuspensions’ potential as Alzheimer’s disease treatment carriers, suggesting that surface modifications or coatings could enhance their targeting efficiency [261].

Nanocrystals, pure drug crystals with no carriers, offer several benefits such as a higher surface-to-volume ratio, an enhanced dissolution rate, and versatile administration routes, ultimately leading to improved bioavailability and therapeutic effectiveness [262,263]. Paeoniflorin, a neuroprotective agent with poor oral bioavailability, was transformed into nanocrystals by Wu et al. using the anti-solvent precipitation method. The nanocrystals exhibited an average size of 139.6 ± 1.3 nm with a zeta potential of −23.2 ± 0.529 mV. In vitro studies demonstrated enhanced release and brain uptake, along with neuroprotective effects on damaged SHSY5Y cells mediated by MPP+ [264]. Stahr et al. highlighted the importance of nanocrystal size in targeting efficiency. Hesperidin nanocrystals of varying sizes were developed, indicating that a smaller size (<200 nm) improved their dissolution rate and solubility, while surface modifications with ligands further enhanced their targeted delivery [265].

Zhu et al. (2021) introduced a nanocrystal-based hydrogel to increase the solubility and permeation of armodafinil, known for cognitive enhancement. Utilizing PVP-K90 and lecithin, they incorporated armodafinil nanocrystals into the hydrogel. This formulation exhibited a high stability due to intermolecular hydrogen bonding. A pharmacokinetic analysis indicated significantly higher brain concentrations with the intranasal nanocrystal-based hydrogel (Cmax = 9533.0 ± 2327.9 ng/mL, Tmax = 0.21 ± 0.08 h) compared to those of its oral administration (Cmax = 4170.0 ± 388.3 ng/mL, Tmax = 0.25 ± 0.00 h). The relative brain-targeting index was 1.99, reflecting the hydrogel’s enhanced brain-targeting ability [266].

Quantum dots (QDs), semiconductor nanocrystals with unique optical and electronic properties, offer a superior stability and multi-functionality for diagnostics. Gao et al. developed CdSe/ZnS QDs coated with poly (ethylene glycol)-poly(lactic acid) nanoparticles (QDs-NPs) to enhance nasal QD delivery. The details are mentioned in Table 8. By further modifying QDs-NPs with wheat germ agglutinin (WGA), they created a WGA-QDs-NP system. Biodistribution studies showed fluorescence signals in the brain region, indicating effective nasal delivery. Fomicheva et al. demonstrated the potential of QDs in detecting Alzheimer’s disease biomarkers, while Thakur et al. explored the capacity of QDs to influence fibrillation [234,267,268]. Diverse nanocarriers, from nano suspensions to nanocrystals and quantum dots, hold promise for enhancing Alzheimer’s disease treatment, offering solutions to challenges such as poor solubility and targeting efficacy.

Dendrimers represent advanced nano-scale systems featuring three-dimensional polymeric cores, which can be tailored for a range of applications. Their capability to traverse cellular membranes, including the blood–brain barrier, has rendered them increasingly valuable in the realm of drug delivery. Notable types of dendrimers encompass poly(amidoamine), PEGylation, pH-sensitive, and peptide dendrimers [276]. Specifically, poly(amidoamine) (PAMAM), polypropylene polybenzylisocyanate (PPI), polylysine (PLL), and carbosilane dendrimers are frequently harnessed for brain targeting.

Dendrimers like Poly(amidoamine) (PAMAM), Polypropylene polybenzylisocyanate (PPI), Polylysine (PLL), and carbosilane are commonly utilized for brain targeting owing to their branched structure, which facilitates the functionalization of active agents and ligand-like peptides derived from ApoE. This enhancement in recognition by LDL receptors present on the endothelial cells of the central nervous system leads to improved uptake and targeting [277]. To overcome the limited half-life and suboptimal permeability of flurbiprofen across the blood–brain barrier, Al-azzawi et al. devised flurbiprofen dendrimers using the solid-phase peptide method for Alzheimer’s disease treatment. The synthesized dendrons loaded with FP were successfully characterized via mass spectrometry and FTIR. Their biocompatibility was evaluated through cytotoxicity assays, and a notable level of permeability (14.79 ± 2.06) was observed for an in vitro model of the BBB as analysed via HPLC [278].

### 4.5. InSitu Gelling System

In-situ gels can be classified into various types based on the external stimuli employed, including temperature-sensitive, ion-sensitive, and pH-sensitive gelling systems. These formulations undergo a transformation from a sol to a gel state in the presence of specific external triggers. In the context of intranasal drug delivery, in-situ nasal gels offer several advantages, such as an extended residence time, enhanced drug penetration, increased payload capacity, improved elasticity, and robust stability due to the gels’ crosslinking property. Moreover, they enable sustained drug release, as elucidated by Hamano et al. in 2018 [123]. Agrawal et al. (2020) elucidated the existence of diverse types of in-situ gelling systems, encompassing temperature-sensitive and ionic cross-linking systems. Depending on the specific type of in-situ gel, various polymers such as xyloglucan, EHEC, pluronic, poloxamer, carbopol, gellan gum, and chitosan are employed [279]. In efforts to mitigate the side effects associated with oral administration.

Patil et al. devised a mucoadhesive in-situ gel incorporating cubosomes containing donepezil. The cubosomes were formulated using glycerol mono-oleate and poloxamer 407, optimized through a central composite design. The optimum cubosome formulation comprised 2% glyceryl mono-oleate and 1.5% poloxamer 407. Additionally, gellan gum (0.3%) and konjac gum (0.03%) were utilized as the gel-forming and mucoadhesive components, respectively. The optimized in-situ gel underwent characterization for various parameters, encompassing the zeta potential, size, PDI, and % EE. The prepared cubosome-based in-situ gel exhibited a drug content of 90.16 ± 1.02%, along with a pH of 6.4 ± 1.29. Notably, the viscosity of the cubosome-based in-situ gel was measured at 180 ± 9.5 cps, accompanied by a gel strength of 34 ± 2.11 s. The in vitro drug release showcased an initial burst release of 24.52% at 2 h, followed by 53.73% at the end of 6 h. Biodistribution studies conducted in vivo exhibited the highest CMAX values for the brain with the cubosome-based in-situ gel (24.01 ± 7.32 µg/mL), followed by the cubosome dispersion (14.34 ± 6.31 µg/mL) and the plain drug solution (3.96 ± 2.38 µg/mL). The Tmax (minutes) value of the dispersion and in-situ gel was 60 ± 0.0. The AUC (0–240 min) for the in-situ gel was 2460.19 ± 4.42 (µg·min·mL^−1^), whereas for the plain dispersion, it was 2002.55 ± 5.56 (µg·min·mL^−1^). Thus, the in-situ gel demonstrated enhanced brain targeting via the nasal route [280].

Cunha and colleagues developed an in-situ gel loaded with rivastigmine (RVG) employing nanoemulsion and nanostructured lipid carriers (NLCs) to prolong the residence time within the nasal cavity. Through meticulous optimization involving different percentages of the thermosensitive polymer, the final batch containing 17% Kolliphor^®^ P407 and 0.3% MethocelTM K4M was identified as the most effective. The RVG-loaded nanoemulsion exhibited a particle size of 141.70 ± 0.40 nm with a PDI 0.45 ± 0.00, while the RVG-loaded NLCs possessed a particle size of 146.10 ± 1.73 nm with a PDI 0.43 ± 0.02. A texture analysis revealed that the NLC-loaded gel demonstrated superior firmness and bioadhesion compared to the nanoemulsion-based gel. Both formulations exhibited enhanced firmness and adhesiveness compared to the plain gel, indicating the potential of nanosystem-based in-situ gels to enhance the retention time within the nasal cavity [281].

Chen et al. pioneered the development of a dual-responsive (pH and temperature) in-situ gel of huperzine A using chitosan as a pH-responsive mucoadhesive polymer and pluronic F127 as a temperature-sensitive agent. This gel was designed to address challenges associated with low bioavailability and efficacy. The hup A microemulsion (ME) and hup AME temperature- and pH-sensitive in-situ gel (TPISG) demonstrated an average size of 21.26 nm and 20.53 nm, respectively. The optimized hup AME TPISG formulation exhibited a clear, free-flowing liquid state at room temperature (560 ±10 mPa s), transitioning to a highly viscous gel (5200 ± 100 mPa s) under nasal conditions. The gelation time was 89 s, with a gelation temperature of 29–34°C. Invitro studies comparing different formulations with initial burst releases (hup AME: 10.7%, hup A solution: 8.8%, hup AMTISG: 9.0%, and optimized hup AMETPISG: 10.6%) over a 0.5h period revealed sustained release in the case of the optimized hup AMETPISG, with 90.52% release at 24 h, attributable to the presence of pluronic F127. An in-vivo evaluation using the microdialysis method indicated improved brain targeting and patient compliance (Table 9).

## 5. Toxicity and Safety Aspects of Nanoparticulate Delivery

Nanoparticles possess the potential to introduce toxicity at various levels, ranging from organs, tissues, and cells to even subcellular components, owing to their distinct physicochemical attributes [288,289]. Table 10 provides detailed preview of detailed toxicity studies conducted on plethora of nanomaterials. Certain metal particles have demonstrated heightened toxicity as their size diminishes, despite their inert nature. Nanoparticles engage with enzymes and proteins within cells, disrupting antioxidant defence mechanisms, leading to the generation of reactive oxygen species and eliciting inflammatory responses, ultimately resulting in necrosis [290]. The toxicity associated with nanoparticles is contingent upon a spectrum of physicochemical factors, including dose, size, surface area, concentration, crystalline structure, aspect ratio, surface coating, and functionalization [291], as well as chemical stability [292,293]. Given that industrial nanoparticles predominantly comprise heavy metals, the compatibility and toxicity factors warrant careful consideration. Although the bioavailability of heavy metal NPs may be restricted, their inherent toxicity remains substantial [292]. For instance, superparamagnetic iron oxide NPs have been documented to modulate gene expression, cellular response, homeostasis, and even cell cycle dynamics [294]. While an abundance of research is dedicated to unravelling the impact of nanoparticle toxicity on human health, some studies have delved into the ecological ramifications to foster the sustainable utilization of this innovative material [290].

## 6. Regulatory Aspects/Challenges of Intranasal Nanocarrier Drug Delivery

Despite the emergence of numerous approved nanomedicines in recent decades, many countries still lack well-defined regulations governing the marketing and utilization of nanocarrier-based formulations. This regulatory gap has constrained the full clinical potential of nanomedicines, underscoring the urgency of collaborative initiatives among global regulatory bodies to establish a robust framework for nanocarrier development. In the absence of explicit guidelines, certain assessments related to the safety, toxicity, and compatibility of nanoformulations are conducted following strategies akin to those employed for conventional therapies [298,299].

The regulation of biologics-based nanomedicines falls within the purview of the framework devised by the European Medicines Agency (EMA). For formulations encompassing proteins and antibodies, the manufacturer is mandated to adhere to the regulations governing new chemical entities (NCEs) and biological medicinal products [300,301]. Conversely, the EMA employs case-by-case analyses for non-biological complex drugs (NBCDs). In specific scenarios, regulatory guidelines for NBCDs can align with the biological framework [302].

The development of nanomedicines presents a substantial challenge owing to the necessity for an extensive characterization of their attributes, which can be easily influenced by even minor modifications. Researchers have been actively pursuing targeted drug delivery through ligand attachment, receptor engagement, or conjugation with diagnostic imaging agents. In such instances, novel approaches are required to assess their physicochemical properties and performance, encompassing considerations such as biocompatibility, protein interactions, and drug metabolism, among others [303].

In India, the requisites for quality, safety, and efficacy data differ based on the approval status of drugs and nanocarriers. All nanopharmaceutical formulations are treated as Investigational New Drugs (INDs), yet their scrutiny may vary depending on diverse categories. If both the drug and nanocarrier are novel and lack prior approval, they are treated as an IND, necessitating adherence to Schedule Y of the Drug and Cosmetics Rule, 1945. Similar guidelines apply to approved nanocarriers paired with new drugs, albeit independent studies specific to nanocarriers may not be mandatory. For fresh nanocarriers paired with traditional or conventional drugs, complete adherence to the Schedule Y IND guidelines might not be requisite, but documented evidence of safety and efficacy remains imperative. In cases in which both the drug and nanocarrier have been previously sanctioned, abbreviated studies are undertaken. The data requisites for nanopharmaceuticals are approached on a case-by-case basis, factoring parameters such as their biological and physicochemical attributes, alongside other considerations, such as the available data for the drug or nanocarrier, encompassing nonclinical proof of concept to clinical challenges.

The FDA and EMA have formulated evaluation guidelines for intranasal formulations, encompassing diverse factors like physical characterization, plume geometry, resting time effects, agitation requirements, particle size distribution, photo-stability, and microbial challenges. The specific tests mandated might vary based on factors such as the formulation type (e.g., suspension, drops, or powder), the device employed (e.g., metered dose container), and the intended application (e.g., single or multiple sprays). While the FDA and EMA have provided suitable methodologies for conducting these evaluations, there exists a scarcity of comprehensive procedural guidelines for their execution. Despite significant strides in nanomedicine-based drug delivery systems, their scaling up and advancement have encountered obstacles due to the absence of universally accepted and harmonized regulatory directives for their assessment and process control. The current intricate and sophisticated nanostructured formulations present additional regulatory complexities [299]. To surmount these challenges, regulatory agencies must collaborate to establish a universally recognized protocol for intranasal nanocarrier systems. This protocol should encompass comprehensive evaluation guidelines and also encompass considerations for process-related variables that might impact the final performance of the product.

## 7. Conclusions

Intranasal drug delivery utilizing nanotechnology has emerged as a promising approach for addressing the challenges posed by Alzheimer’s disease management. The convergence of nanotechnology and pharmaceutical sciences has yielded innovative strategies to enhance the efficacy, bioavailability, and targeted delivery of therapeutic agents across the blood–brain barrier. This review has explored the substantial progress made in this field, shedding light on the advancements, challenges, and future prospects for intranasal drug delivery in Alzheimer’s disease management. The utilization of nanocarriers, such as liposomes, solid lipid nanoparticles, nanoemulsions, and dendrimers, has enabled the precise encapsulation and controlled release of Alzheimer’s disease therapeutics. These nanocarriers offer improved drug stability, a prolonged residence time, and the potential for targeted brain delivery. Furthermore, their versatile nature allows for surface modification, ligand conjugation, and multi-functionalization, thereby enhancing their ability to cross biological barriers and interact with specific cellular receptors. While the potential of intranasal nanocarrier drug delivery for Alzheimer’s disease treatment is significant, several challenges warrant attention. Toxicity and safety concerns associated with nanomaterials necessitate comprehensive evaluations and standardized regulatory guidelines to ensure patient safety. Additionally, the scalability of nanotechnology-based formulations remains a pivotal concern, as their transition from laboratory research to large-scale production requires rigorous optimization and cost-effectiveness’s, the multi-facet pathology of AD poses a significant challenge in the clinical translation of ongoing research. There still exists a gap in our understanding of the etiology and identification of potential targets. Based on this understanding, small-molecule and associated formulations can be developed. Thus, fostering inter-disciplinary collaborations among researchers, clinicians, and regulatory bodies could provide valuable insights for tackling these problems.

## 8. Future Prospects

The future trajectory of intranasal drug delivery for Alzheimer’s disease management via nanotechnology is imbued with profound promise. To harness this potential to its fullest extent, it is essential to foster collaborations among researchers, clinicians, and regulatory agencies. The following avenues hold considerable promise for advancement:

Precision Targeting: Delving into advanced targeting strategies involving ligands, peptides, or biomolecules could yield precise and potent brain delivery. Tailoring nanocarriers to selectively engage relevant receptors promises to amplify their therapeutic efficacy.

Personalized Therapies: By harnessing patient-specific nanomedicines, the treatment landscape could undergo a paradigm shift. Accounting for individual genetic and physiological nuances through tailored therapies could optimize outcomes and curtail adverse effects.

Theranostic Platforms: Orchestrating diagnostic and therapeutic functionalities within a singular nanocarrier configuration can furnish real-time insights into drug delivery and treatment response. This integrated theranostic approach could furnish personalized treatment paradigms.

Regulatory Framework Enhancement: Crafting comprehensive, well-defined regulatory guidelines tailored to the realm of nanomedicine-based intranasal drug delivery is of paramount importance. Such harmonized regulations on a global scale are instrumental in the seamless translation of research findings into clinical practice.

Biomarker Integration: Enmeshing biomarker insights into the early diagnosis and monitoring of Alzheimer’s disease could be transformative. The nanotechnology-enabled intranasal delivery of diagnostic agents might herald more accurate disease assessments.

Combination Therapies: Leveraging the versatility of nanocarriers to facilitate the co-delivery of multiple therapeutic agents presents an avenue to harness synergistic effects and tackle the multifaceted nature of Alzheimer’s disease.

Long-Term Safety Endeavours: Rigorous, long-term investigations are indispensable to ensuring the safety and compatibility of nanocarriers, including their potential cumulative effects over extended periods.

In summary, the integration of nanotechnology into intranasal drug delivery opens up new possibilities for effective Alzheimer’s disease management. While challenges exist, collaborative efforts among researchers, clinicians, and regulatory authorities are pivotal in realizing the full potential of this ground-breaking approach. By skillfully addressing these challenges and moving towards future prospects, intranasal nanotechnology-based therapies may usher in a transformative era characterized by personalized, effective, and safer interventions for Alzheimer’s disease.

## Figures and Tables

**Table 1:** List of monoclonal antibody-based immunotherapies explored for targeting different hallmarks of AD (e.g., Aβ and tau proteins, etc.).

Name	Status	Outcome	Ref
Donanemab	Phase 3	Donanemab showed maximum affinity towards Aβplaques, resulting in a deceleration of disease progression. Further, PET images revealed the absence of Aβ plaques in patients after12 months of treatment.	[58]
Bapineuzumab	Failed in Phase 3	Had an anti-Alzheimer’s effect by targeting tau phosphorylation, thereby decreasing the tau concentration in CSF. However, bapineuzumab failed to show clinical efficacy and its clinical use was associated with a high risk of ARIA and TEAE.	[59]
Solanezumab	Terminated	Solanezumab acts by identifying and targeting soluble monomer Aβ except for fibrillary Aβ. However, the trial was terminated due to negligible benefits to mild AD patients and not meeting clinical endpoints.	[60]
Crenezumab	Completed	This antibody was well tolerated with no prominent side effects even when increasing the dosage. However, no commercial translation occurred as it failed to show clinical efficacy.	[61]
Ponezumab	Phase 2	Treatment with ponezumab led to increased Aβ level in plasma. On completion of treatment, no alterations in CSF biomarkers, Aβ burden, and cognition were reported, which could be due to its low penetrability.	[62]
Semorinemab	Phase 2	Semorinemab had a well-tolerated safety profile. The 73-week treatment did not reduce disease progression and no clinical outcome was reported.	[63]
Gosuranemab	Phase 2	Considerable concentration of gosuranemab in serum and CSF was noted. Further, ~98% of unbound tau was reduced in CSF, yet no benefits were observed in a population at risk for PSP.	[64]
Tilavonemab	Phase 2	Tilavonemab showed no effect on disease progression. Intriguingly, a reduced level of free tau in CSF (38.0–46.3%) was reported, which reached a plateau when the dosage was increased.	[65]

**Table 2:** Overview of various polymers that have been investigated for delivering therapeutics targeting Alzheimer’s disease (AD).

Drug	Polymer	Targeting	Route of Administration	Results	Ref
Estradiol	Polylactide-co-glycolide (PLGA)	Tween 80 (mimics LDL particles by adsorbing apolipoprotein and achieves targeting via LDL receptors)	Oral Route	Higher brain uptake (1.969 ± 0.197 ng/g) was seen in coated NPs compared to that of uncoated PLGA nanoparticles (1.105 ± 0.136 ng/g) Enhanced drug fraction reached brain following oral administration.	[94]
Donepezil	PLGA-*b*-PEG	-	NA	Donepezil nanoparticles demonstrated destabilization action against Aβ fibril. Improved transport across in vitro BBB model as compared to free drug	[95]
Donepezil	Polylactide-co-glycolide (PLGA)	Tween-80 (internalize via LDL receptors)	Intravenous	Biphasic release pattern with sustained release (87.42 ± 0.06%) for up to 25 days Higher Cmax in brain homogenate (121.68 ± 13.23 ng/mL) as compared to that of drug solution (6.66 ± 1.13 ng/mL)	[96]
Rivastigmine	Chitosan	Tween-80 (internalize through LDL receptors)	Intravenous	Chitosan NPs demonstrated high drug loading (11.51 ± 0.32%) with particle size of 47± 4 nm Biphasic release pattern with sustained release (97.25 ± 0.83%) for up to 12 h. Reduced accumulation in liver, spleen, and heart.	[97]

**Table 3:** List of various lipid carriers, including liposomes, solid lipid nanoparticles (SLNs), nanostructured lipid carriers (NLCs), and nanoemulsions, that have been investigated for delivering therapeutics targeting Alzheimer’s disease (AD).

Drug	Carrier	Target	Route	Description	Ref
Curcumin/Ginsenoside Rb1	Liposome	Mannose	i.v.	Demonstrated high encapsulation efficiency for mannose–curcumin (94.23 ± 2.886) % and Rb1 (90.56 ± 1.307) % with particle size of approx. 100 nm Dual-loaded liposomes exhibited increased cell uptake and accumulation in N2a cells In vivo studies in APP/PS-1 mice showed reduced oxidative stress and inflammation	[102]
miR-101 and Curcumin	Liposome	-	NA	miR-101 liposomes demonstrated lowering of Aβ for up to 3 h. Prolong effect was seen for 12 h when using a combination approach Curcumin demonstrated delayed and indirect effects on mRNA^APP^ transcription Meanwhile, miR-101 shows a direct and rapid effect on transcription Further prepared dual liposomes demonstrated an anti-inflammatory effect	[103]
Caffeic Acid	Liposome	Transferrin	NA	The Tf-CA-liposomes had size of 139 ± 9 nm with PdI of 0.20 ± 0.03 and %EE of 23 ± 4% The modification with Tf was confirmed with ATR-FTIR The sustained release was observed with 11 ± 3% at 24 h ThT fluorescence showed disaggregation capacity of Tf-CA liposome against Aβ42 peptide in which 13% reduction in fibril was observed after 1 h of incubation	[104]
Memantine HCl and Tramiprosate	SLN	-	oral	Tramiprosate demonstrated higher inhibition (16.56%) in ThT studies as compared to memantine HCl (3.22%) PK studies showed delayed clearance (>4 h) of SLNs as compared to drug solutions (1 h). Also, SLNs showed higher conc. in the brain (177.959 ± 18.366 and 30.294± 2.012 µg/mL) as compared to a solution with a lower concentration in other organs, e.g., liver and kidney PD and behavioral studies indicated a neuroprotective role of SLNs	[105]
Erythropoietin	SLN	-	i.p.	EPO-SLN had an optimum particle size (219.9 ± 15.6 nm), PDI (0.18 ± 0.03), and drug loading (41.4 ± 3.6 IU/mg) In MWM test, EPO-SLNs demonstrated improvement in spatial and learning memory. Histopathological examination showed SLNs’ potential ability to hinder Aβ effects Further reduction in lipid peroxidation was observed in EPO-SLN group	[106]
Berberine	NLC	-	oral	Berb-NLCs were optimized by 3^2^ full factorial model in which final batch had size of 186 nm and 88% EE The Berb-NLCs exhibited sustained release (86%) for up to 24 h Pharmacodynamics studies involving behavioural evaluation showed improved cognition as compared to that when using pure berberine	[107]
Thymoquinone	NE	-	oral	Exhibited decrease Aβ40 and Aβ42 levels in HFCD-induced rats Attenuation in IDE and LRP1 levels was observed which could lead to Aβ degradation	[108]

**Table 4:** A list of research on metal-nanoparticle-based delivery for the treatment of Alzheimer’s disease.

Drug	Carrier	Target	Route of Administration	Description	Ref
-	Myco-fabricated ZnO nanoparticles	-	i.p.	Myco-fabricated ZnO-NPs exhibited substantial anti-inflammatory and anti-acetylcholinesterase properties A therapeutic dose of 5 mg/kg improves learning and memory activity	[141]
PEG-MIL-101 (MOF)	AuNPs			The PEG-MIL-101 conjugated anionic AuNPs exhibited uniform binding with Aβ monomers and Aβ_42_ fibrils Developed PEG-MIL 101-AuNPs demonstrated a marked decline in fibrillation by disrupting Aβ_42_ fibrils, thereby decreasing the aggregation	[142]
-	Cadmium sulfide and Iron oxide nanoparticles	Protein capped	NA	The PC-CdS (≤20 nm) and Fe3O4 NPs (~40–50 nm) had a nanometric size Concentration-dependent and time-dependent tau inhibitory action was exhibited by protein-capped CdS (63%) and Fe3O4 (49%) NPs Upon treatment with NPs, a significant decrease in fibrillary aggregation was observed	[143]
Rhein and Polydopamine	Fe–Rh/Pda NPs	(KLVFFAED)/K8 peptide	i.v.	The 7T MRI images showed efficient transit of NPs across BBB Also, SDA-PAGE analysis following treatment revealed considerable Aβ_42_ targeting ability of developed NPs, which was further verified by in vivo studies in APP/PS1 mice Prepared NPs remarkably improved brain bioavailability (~11.2-fold) of rhein as compared to that of rhein solution Improved antioxidant and anti-Aβ effects were reported	[144]
	Ruthenium dioxide	Borneol	i.v.	RuO_2_-Bor showed concentration-dependent enzymatic activity including CAT, SOD, and POD Significant decrease in ROS level was observed indicating ROS scavenging action RuO_2_-Bor NPs exhibited inhibitory action on Aβ_42_ aggregation (~18.8%), maintained mitochondrial homeostasis, and restored cognition function in Aβ_42_ mice	[145]

**Table 5:** Overview of research on nanoparticle-aided intranasal delivery of anti-Alzheimer’s drugs.

Drug	Nanoparticle	Targeting Agent	Method of Preparation	Pharmacological Data	Ref
Tacrine	poly (n-butyl cyanoacrylate)	polysorbate 80	Emulsion polymerization technique	In comparison to uncoated nanoparticles and free tacrine, a substantially increased tacrine concentration (170 ng/mL) was observed in the brain upon coating poly(n-butylcyanoacrylate) nanoparticles with 1% polysorbate 80.	[227]
Galantamine	Hydrobromide Chitosan complex NP (GH–chitosan NP)	-	Ionic interaction method	Prolonged release was obtained (58.07% ± 6.67 after 72 h) with delayed mucociliary clearance GH-chitosan NPs showed improved cholinergic activity with reduced AchE levels No significant toxicity to the brain was observed	[228]
Estradiol	Chitosan NP		Ionic interaction method	NPs loaded with estradiol showed significantly lower concentration in plasma i.n. (32.7+/− 10.1 ng mL^−1^; t(max) 28 +/− 4.5 min) as compared to i.v. (151.4 +/− 28.2 ng mL^−1^) Higher concentration(76.4 +/− 14.0 ng mL^−1^ and t(max) 28 +/− 17.9 min) in CSF were observed for i.n. delivery as compared to those with i.v. delivery	[229]
-Gene (DNA)	Polyamidoamine dendrimers-Polyethlene glycol (PAMAM-PEG-) NP	Angiopep	First, PEG PAMAM modification of angiopep was performed followed by complexation with DNA	Higher efficiency to penetrate and accumulate in the brain was observed with angiopep-modified NPs as compared to non-modified NPs with higher gene expression	[230]
Doxorubicin	Stealth(PEG2000) and non-stealth SLN		High-pressure homogenization	An increased accumulation of doxorubicin was observed in the brain upon increasing the level of stealthing agent PEG2000 Amount of doxorubicin in the brain after 30 min was found to be 27.5 ng/g in case of nonstealth SLNs while it was 242.0 ng/g for stealth SLNs loaded with 0.45% PEG; this pattern persisted for 2 h	[231]
RVG-9R -BACE1 siRNA A	Chitosan-coated and uncoated SLN	-	High-pressure homogenization	For siRNA, a 15 min lag time was reported whereas it took 30 min using NPs coated with chitosan	[232]
Curcumin	Lipid NP	-	Hot solvent diffusion method	Curcumin lipid NPs showed sustained release upto 72 h The DPPH assay demonstrated 95% scavenging activity It also showed enhanced permeation as compared to the free curcumin Cytotoxicity studies demonstrated no toxicity with GI50 >80g/mL	[233]
Vasoactive intestinal peptide (VIP)	PEG-PLA NP	Wheat germ agglutinin	Double-emulsion solvent evaporation	AUC of WGA-VIP NP depicted a more than five-fold increase in the brain uptake upon i.n. administration than plain VIP solution Improved brain delivery (30–50%) was observed for targeted NPs	[234]
Neuroprotective peptide	PEG-co-PCL NP	Lactoferrin	Emulsion solvent evaporation	Enhanced cellular accumulation was observed for lactoferrin-modified NPs as compared to unmodified NPs The AUC of Coumarin-6-incorporated lactoferrinNPs was 1.56 fold higher in olfactory tract than the Coumarin-6-incorporated unmodified NPs	[235]

**Table 6:** List of different liposome-based intranasal drug delivery systems explored for Alzheimer’s disease treatment.

Liposome Formulation	Problem to Encounter	Pharmacological Data	Ref
Bifunctionalized liposome mApoE-PA-LIP	Effective targeting of Aβ	The mApoE-PA-LIP showed temporal and dose-dependent inhibition of Aβ_42_ aggregates, while destabilization of preformed aggregates was found to be time- and lipid-dose-dependent. Also, five-fold increased radioactivity of brain/blood ratio was seen for mApoE-PA-LIP compared to PA-LIP.	[236]
Transferrin-modified alpha-M liposomes	Poor penetration	The alpha-M demonstrated an entrapment efficiency greater than 88% with improved bioavailability	[237]
Fluorescent liposomes functionalized with Antibody R17217	Effective binding to Aβ	Functionalization improved cellular uptake and permeation. The functionalized liposomes also demonstrated higher EP (7.24 ± 0.39 ×10^−6^ cm/min) as compared to that of biotin/streptavidin-RI-A-LIP (4.97 ± 0.51 × 10^−6^ cm/min).	[238]
Multifunctionalized liposomes attached with two BBB-specific ligands and curcumin–lipid ligand	To locate and target formulation	In vivo study in mice demonstrated efficacy of liposomes to traverse across BBB. Addition of TREG–lipid curcumin derivative in liposome did not influence the functionality of ligands	[239]
Liposome coated with chitosan and encapsulated with fexofenadine	Effective brain targeting	Increased stability and retention time. Chitosan-coated liposomes showed enhanced bioavailability (34.7 ± 6.3%) as compared to non-liposomes (25.0 ± 8.0%) and uncoated liposomes (24.5 ± 7.5%). Sustained release was obtained for a period of 12 h	[240]

**Table 7:** A list of various research studies that explored the potential of microemulsion and nanoemulsion delivery systems for Alzheimer’s disease treatment.

Drug and DDS	Pharmacological Evidence	Ref
Risperidone-loaded chitosan-based nanoemulsion	The mucoadhesive nanoemulsion was most effective with higher drug targeting efficiency (476 ± 2.14%) and rapid transport as compared to the drug solution	[254]
Saquinavir mesylate-loaded nanoemulsion	A higher concentration of drug (7290.46± 143.15 ng/g) was found at a faster rate with the NE with no toxicity and higher targeting efficiency (2919.261 ± 5.68%)	[255]
Pomegranate seed oil (PSO) nanoemulsion	PSO contains phytoconstituents such as polyunsaturated fatty acids and punicic acid, which reduced lipid oxidation and loss of neuronal functionality, suggesting the formulation to be neuro-protective and safe	[256]
Curcumin-based o/w nanoemulsion	Curcumin has low solubility and poor bioavailability. To improve its bioavailability, a curcumin-loaded NE was formulated. The prepared NE had a droplet size in the range of 618.6 nm to 79.5 nm. Anti-inflammatory action was shown using mouse ear inflammation model induced by TPA. The inhibition percentages observed were43% (in case of 618.6 nm droplets) and 85% (79.5 nm droplets), respectively.	[257]
anti-TNFα siRNA-encapsulated flaxseed nanoemulsion	SiRNA-loaded nanoemulsion showed 70 ± 10% encapsulation efficiency. Higher cellular uptake was observed at 15 min end point (10-fold greater) and after 2.5 h (25-fold greater), respectively. Nanoemulsion loaded with SiRNA showed improved brain targeting (two-fold greater) than SiRNA solution at the end point of 6 hr. Nanoemulsion-based delivery was found to be effective in gene knockdown and preventing neuroinflammation	[258]

**Table 8:** A list of various studies that investigated quantum dots as a suitable carrier for delivery of different anti-AD therapeutics via intranasal route.

Drug and Carrier	Investigation	Results	Ref
PEG-BTA quantum dots	Specificity and sensitivity of disease detection	Effective binding to amyloid beta peptide	[269]
Graphene QDs	Inhibitory effect on Aβ	Suppressed formation of fibrils. The inhibitory effect increased when surface negative charge decreased	[270]
Curcumin–graphene QD coated with Indium-TO electrode	For detection of ApoE4	Reproducibility, repeatability, and high efficiency of curcumin platform for sensing even in a complex matrix	[271]
High-fluorescence NGQDs	To sense enzymatic action and efficacy	Decreased activity of AChE	[272]
Biotinylated N-Ab and streptavidinquantum dots	To detect Aβ	Successful for detecting Aβ in CSF	[273]
N-acetyl-L-cysteine-capped quantum dots	For inhibition of amyloid fibrillation	Inhibitory effect with an AUC that was 100 times increased	[274]
Grapheme quantum dots (GQDs) conjugated with peptide glycine–proline–glutamate	Neuroprotective effect	Inhibition of fibril with enhanced memory and reduced inflammation	[275]

**Table 9:** Summarizes various attempts by researchers to improve brain targeting using an insitu gelling system.

Drug	Pharmacological Data	Ref
Poly (N-vinyl pyrrolidone) functionalized insulin nanogel	Receptor binding with protection from degradation and effective transport	[282]
E-beam-irradiation-based nanogel of poly(N-vinyl pyrrolidone) attached to insulin	Intranasal delivery was enhanced based on activated level of AKT with increased insulin delivery	[283]
Donepezil nanogel functionalized with Poly(N-isopropylacrylamide) (PNIPAM)	Biocompatible with sustained release pattern and enhanced entrapment efficiency of 87.5%	[284]
Methotrexate nanogels coated with polysorbate 80	Effective brain targeting was achieved by coating with polysorbate 80	[285]
Cholesterol-modified pullulan (CHP)- loaded hydrogel nanoparticles	Interacted with oligomeric Aβ and reduced its toxicity	[286]
Oligonucleotide-based nanogel	Less degradation with 15-fold enhanced biodistribution and two times less accumulation in the liver as compared to naked ODN	[287]

**Table 10:** Lists various toxicity studies conducted using diverse types of nanomaterials and their pharmacological inferences.

Nanomaterial	Pharmacological Data	Ref
Surface-modified gold NPs of various sizes	The concentration of gold atoms up to ~100µM does not cause any toxicity to leukemia cells. Cell viability studies demonstrated no cytotoxicity	[294]
Engineered gold NPs	NPs with a diameter of1–2 nm showed toxicity due to irreversible binding. No toxicity was observed in the case of NPs of the range of3–100 nm	[142]
Metal oxide NPs (TiO_2_, ZnO, FeSO_4_, Al_2_O_3_, and CrO) with a size range of 30–45 nm	FeSO_4_, Al_2_O_3_, and TiO_2_(concentration > 200 µg/mL) demonstrated no toxicity and at high doses, they showed LDH leakage. ZnO with a concentration range of 50–100µg/mL reduced mitochondrial function	[295]
Silver NPs	NPs showed toxicity via oxidative stress and a concentration of 5–50µg/mL reduced mitochondrial function along with enhanced LDH leakage	[293]
Silver NPs with surface charges	AgNPs exhibited toxicity depending upon their surface charge	[296]
Gold, silver, and platinum NPs	Exhibited toxicity via accumulation. Out of all three, the silver NPs were the most toxic whereas gold NPs were non-toxic	[297]

## Data Availability

Data sharing is not applicable.

## Abbreviation

AD	Alzheimer’s disease
CNS	Central nervous system
BBB	Blood–brain barrier
APOE	Apolipoprotein E
PSEN	Presenilin
APP	Amyloid precursor protein
NMDA	N-methyl-D-aspartate receptor
NFT	Neurofibrillary tangles
CS-NP	Chitosan nanoparticles
ChAT	Choline acetyltransferase
PIP	Piperine
PAMAM	Poly (amidoamine)
PVA	Polyvinyl alcohol
PLGA	poly lactic co glycolic acid
HPMC	Hydroxypropyl methylcellulose
i.n	Intranasal
i.v	Intravenous
NP	Nanoparticles
SLN	Solid lipid nanoparticles
NLC	Nanostructured lipid carrier
Lf-TMC-NP	Lactoferrin conjugated N-methylated chitosan nanoparticles
RSLN	Risperidone solid lipid nanoparticles
PDI	Polydispersibility Index
EE	Entrapment efficiency
BuChE	Butyrylcholinesterases
AChEI	Acetylcholinesterase inhibitor
ROS	Reactive oxygen species
ARIA	Amyloid-related imaging abnormality
FDC	Fixed dose combination
OLE	Open label extension
PET	Photon emission topography
PEG	Polytheylene glycol
AMT	Adsorption mediated transcytosis
Tg	Glass transition temperature
PLGA	Polylactide-co-glycolide
LNP	Lipid nanoparticles
MOF	Metal organic framework
MDA	Malonyldialdehyde
IVIVC	In vitro in vivo correlation
RHT	Rivastigmine
TNF	Tumour necrosis factor
ELISA	Enzyme-linked immunosorbent assay
TRAIL	TNF-related apoptosis-inducing ligand
mi-RNA	Micro ribonucleic acid
PLA	Polylactic acid
AuNP	Gold nanoparticles
SPION	Super paramagnetic iron oxide nanoparticles
HAS	Hydroxy-α-sanshool
GH	Galantamine hydroxide
OVAL	Ovalalbumin
AUC	Area under the curve
SNF	Simulated nasal fluid
ACSF	Artificial cerebrospinal fluid
PBS	Phosphate buffer saline
DPPH	2,2-diphenyl-1-picrylhydrazyl
FRAP	Ferric reducing ability of plasma
NE	Nanoemulsion
GQR	G alpha subunits
DTE	Drug transport efficiency
DTP	Drug targeting potential
MPP	1-Methyl-4-phenylpyridinium
QD	Quantum dots
WGA	Wheat germ agglutinin
ApoE4	Apolipoprotein E4
GQD	Graphene quantum dots
FTIR	Fourier transform infrared spectroscopy
HPLC	High-performance liquid chromatography
Cmax	Maximum concentration
EMA	European Medicines Agency
NBCD	Non-biological complex drugs
IND	Investigational new drug
Aβ42	42-amino acidβ amyloid
